# Dipotassium zinc tetra­iodate(V) dihydrate

**DOI:** 10.1107/S1600536810006008

**Published:** 2010-02-20

**Authors:** Jan Fábry, Radmila Krupková, Ivana Císařová

**Affiliations:** a Institute of Physics, Academy of Sciences of the Czech Republic, Na Slovance 2, 182 21 Praha 8, Czech Republic; b Department of Inorganic Chemistry, Faculty of Science, Charles University, Hlavova 8, 128 43 Praha 2, Czech Republic

## Abstract

The title compound, K_2_Zn(IO_3_)_4_·2H_2_O, contains two symmetry-independent K and I atoms. These atoms, as well as the Zn atom, are coordinated by shared O atoms and, moreover, the Zn atom is coordinated by two water mol­ecules in *trans* positions. The K, Zn and water O atoms atoms are situated in special positions on twofold symmetry axes. The hydrogen atoms are involved in strong O—H⋯O hydrogen bonds and O—H⋯I inter­actions also occur. The crystals of the title compound are, in general, twinned, but the sample used for this experiment was free of twinning.

## Related literature

Single crystals of KIO_3_ grown from aqueous solution develop as domained crystals of poor quality but the quality of the crystals obtained can be affected by additional reagents such as HIO_3_, see: Hamid (1974 ▶); Lü & Zhang (1987 ▶). For related structures, see Vinogradov *et al.* (1979 ▶); Maneva & Rabadjieva (1994 ▶); Juncheng *et al.* (2000 ▶); Lepeshkov *et al.* (1977 ▶); Lucas (1984 ▶). For hydrogen bonding, see: Desiraju & Steiner (1999 ▶). For a description of the Cambridge Structural Database, see: Allen (2002 ▶). For the PDF-2 Powder Diffraction Database, see: ICDD (2000 ▶) and for the Inorganic Crystal Structure Database, see: ICSD (2009 ▶). For the extinction correction, see: Becker & Coppens (1974 ▶).

## Experimental

### 

#### Crystal data

K_2_Zn(IO_3_)_4_·2H_2_O
*M* *_r_* = 879.2
Monoclinic, 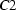

*a* = 13.8044 (3) Å
*b* = 7.7285 (2) Å
*c* = 8.2860 (2) Å
β = 126.5726 (13)°
*V* = 709.95 (3) Å^3^
*Z* = 2
Mo *K*α radiation
μ = 11.08 mm^−1^
*T* = 295 K
0.17 × 0.12 × 0.05 mm

#### Data collection

Nonius KappaCCD area-detector diffractometer
Absorption correction: gaussian (Coppens, 1970 ▶) *T*_min_ = 0.241, *T*_max_ = 0.581
11909 measured reflections
1639 independent reflections
1595 reflections with *I* > 3σ(*I*)
*R*_int_ = 0.045

#### Refinement

*R*[*F* ^2^ > 2σ(*F* ^2^)] = 0.019
*wR*(*F* ^2^) = 0.055
*S* = 1.72
1639 reflections
105 parameters
4 restraints
H atoms treated by a mixture of independent and constrained refinement
Δρ_max_ = 0.60 e Å^−3^
Δρ_min_ = −0.61 e Å^−3^
Absolute structure: Flack (1983 ▶), 761 Friedel pairs
Flack parameter: −0.01 (3)

### 

Data collection: *COLLECT* (Nonius, 2000 ▶) and *HKL* *DENZO* and *SCALEPACK* (Otwinowski & Minor, 1997 ▶); cell refinement: *COLLECT* and *HKL* *DENZO* and *SCALEPACK*; data reduction: *COLLECT* and *HKL* *DENZO* and *SCALEPACK*; program(s) used to solve structure: *SIR97* (Altomare *et al.*, 1999 ▶); program(s) used to refine structure: *JANA2006* (Petříček *et al.*, 2006 ▶); molecular graphics: Spek (2009 ▶); software used to prepare material for publication: *JANA2006*.

## Supplementary Material

Crystal structure: contains datablocks global, I. DOI: 10.1107/S1600536810006008/fj2279sup1.cif

Structure factors: contains datablocks I. DOI: 10.1107/S1600536810006008/fj2279Isup2.hkl

Additional supplementary materials:  crystallographic information; 3D view; checkCIF report

## Figures and Tables

**Table 1:** Hydrogen-bond geometry (Å, °)

*D*—H⋯*A*	*D*—H	H⋯*A*	*D*⋯*A*	*D*—H⋯*A*
O7—H1*O*7⋯O4^i^	0.85 (9)	1.88 (10)	2.672 (6)	155 (5)
O7—H1*O*7^ii^⋯O4^iii^	0.85 (9)	1.88 (10)	2.672 (6)	155 (5)
O8—H1*O*8⋯O3^ii^	0.845 (16)	1.837 (16)	2.610 (4)	151 (4)
O8—H1*O*8⋯I1^ii^	0.845 (16)	2.96 (3)	3.4119 (10)	116 (3)

Symmetry codes: (i) 
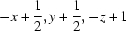
; (ii) 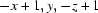; (iii) 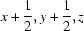.

**Fig. 1.**
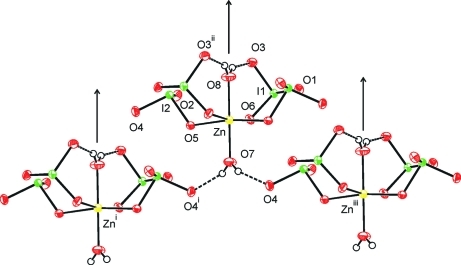
View of the environment of Zn atoms (Spek, 2009) with omission of the K atoms. The displacement ellipsoids are shown at the 50% probability level. The hydrogen bonds are indicated by the dashed lines. The arrows depict two-fold axes. Symmetry code: (i) -x + 1/2, y + 1/2, -z + 1; (ii) -x + 1, y, -z + 1; (iii) -x + 3/2, y + 1/2, -z + 1.

**Fig. 2.**
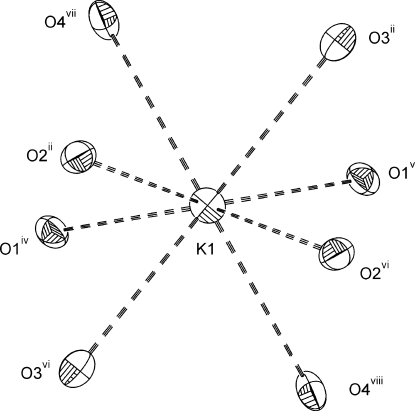
View of of the environment of K1 atom (Spek, 2009). The displacement ellipsoids are shown at the 50% probability level. Symmetry code: as above and (iv) -x + 3/2, y - 1/2, -z + 1; (v) x - 1/2, y - 1/2, z - 1; (vi) x, y, z - 1; (vii) x + 1/2, y - 1/2, z; (viii) -x + 1/2, y - 1/2, -z.

**Fig. 3.**
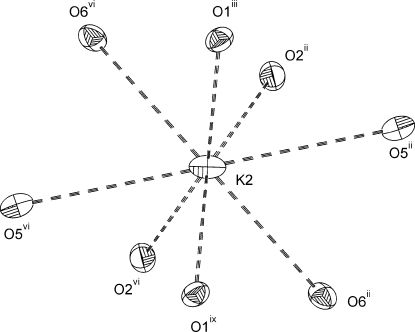
View of of the environment of K2 atom (Spek, 2009). The displacement ellipsoids are shown at the 50% probability level. Symmetry code: as above and (ix) x - 1/2, y + 1/2, z - 1.

**Fig. 4.**
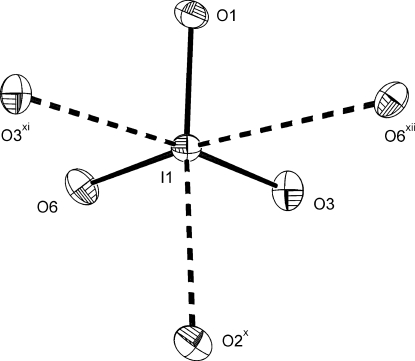
View of of the environment of I1 atom (Spek, 2009). The displacement ellipsoids are shown at the 50% probability level. Symmetry code: as above and (x) -x + 1, y, -z + 2; (xi) -x + 3/2, y + 1/2, -z + 2; (xii) -x + 3/2, y - 1/2, -z + 2.

**Fig. 5.**
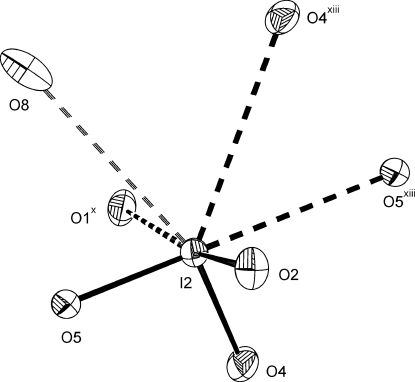
View of of the environment of I2 atom (Spek, 2009). The displacement ellipsoids are shown at the 50% probability level. Symmetry code: as above and (xiii) -x + 1/2, y - 1/2, -z + 1.

## supplementary crystallographic information

### Comment

Motivation for the present structure determination was growth of single crystals of KIO_3_ that develop as domained crystals of poor quality from the water solution only (Hamid, 1974; Lü & Zhang, 1987). However, quality of the obtained crystals can be affected by additional reagents, such as HIO_3_ (Hamid, 1974; Lü & Zhang, 1987). The matter of interest was to find a suitable solution with other additives from which good-quality crystals of KIO_3_ can be obtained.

In this case, the title structure has been grown as it is given in the preparative section.

The compound of the same chemical composition has already been synthesized (Maneva & Rabadjieva, 1994; Juncheng *et al.*, 2000; Vinogradov *et al.*, 1979) along with similar compounds with different central atoms instead of Zn:  According to the powder diffraction experiments, Ni and Co analogoues are isostructural to Zn (Maneva & Rabadjieva, 1994). (However, the powder diffractograms have been given only for the Ni and Co compounds in the latter reference.) Juncheng *et al.* (2000) investigated thermodynamic properties of K_2_ME(IO_3_)_4_.2H_2_O where ME=Mg, Ni and Zn. Lepeshkov *et al.* (1977) studied the systems Zn(IO_3_)_2_ - KIO_3_ - H_2_O as well as Co(IO_3_)_2_ - KIO_3_ - H_2_O at 50°C. The *d* values of K_2_Zn(IO_3_)_4_.2H_2_O obtained from the powder diffraction experiment by Lepeshkov *et al.* (1977) - see also ICDD Card 31-1135, PDF-2 database, ICDD (2000) - fairly correspond to the intensive peaks, that have been calculated from the title structure (Spek, 2009). However, Lepeshkov *et al.* (1977) did not give any details about the conditions of the powder diffraction experiment. Lepeshkov *et al.* (1977) have shown that infrared spectra as well as thermal and differential thermal gravimetric analyses (TGA and DTA) of K_2_Zn(IO_3_)_4_.2H_2_O and K_2_Co(IO_3_)_4_.2H_2_O are similar. The latter authors concluded from the infrared spectra that water molecules as well as four iodates are involved in the coordination sphere of the respective central atoms Zn and Co. Vinogradov *et al.* (1979) further studied systems of ME(IO_3_)_2_ - KIO_3_ - H_2_O, where ME=Co, Mn and Zn. The latter authors confirmed and extended the former findings (Lepeshkov *et al.*, 1977) that the central metal atoms (Co, Mn and Zn) are situated in an octahedron formed by the oxygens stemming from four [IO_3_]^-^ and two H_2_O molecules.

However, the Inorganic Crystal Structure Database (ICSD, 2009) does not contain any structure of the composition given above. Nevertheless, the findings by Lepeshkov *et al.* (1977) as well as by Vinogradov *et al.* (1979) have been confirmed and precised in the present article: The environment of Zn in the title structure is formed by two pairs of symmetry independent iodate groups as well as two independent coordinated water molecules in *trans* positions.

There are several points of interest regarding the title structure. The primitive unit cell (p index) of the title structure can be obtained by the transformation from the centred C cell (C index) [a_p_,b_p_,c_p_]=[//0 -1 0//1/2 -1/2 0//1/2 -1/2 1//][a_C_,b_C_,c_C_]. ([a_p_,b_p_,c_p_] and [a_C_,b_C_,c_C_] are the column matrices while //0 -1 0//1/2 -1/2 0//1/2 -1/2 1// are the first, the second and the third row, respectively, of the 3×3 matrix.) The transformed unit cell parameters are equal to 7.7285 (2), 7.9103 (1), 7.9421 (3) Å, 63.0265 (25), 60.8856 (9), 60.7574 (13)°; V=354.978 (18) Å^3^.

Taking the metric of the unit cell into consideration there is no wonder that twinning has been observed in the title structure, either in the polarization microscope and by preliminary diffraction measurements of several samples that have shown rather broad peaks. However, it seems that the twinning is less severe in the title structure than in KIO_3_. The single-domained crystals can be easily obtained mechanically. Observation of the crystals in the microscope did not show ferroelastic switching of the domains.

The volume of the primitive unit cell as well as lengths of the primitive unit cell axes of the title structure are comparable to those of KIO_3_ that easily forms twins: 7.7436 (4), 7.7183 (4), 7.7328 (5) Å, 108.986 (4), 109.449 (4), 109.209 (5)°; V=359.11Å^3^ - Lucas (1984).

In the title structure, the I atoms are surrounded by a highly distorted oxygen environment, each I is bonded to three oxygens that are substantially closer. In the case of I1 the other three oxygens including the former ones form a distorted octahedron around I1 (Fig. 4) while in the case of I2 there are four more distant oxygens completing the coordination of the latter atom (Fig. 5). The environments of the iodines (Figs. 4 and 5) are rather similar to that in KIO_3_ where are also 3 oxygens substantially closer to the central I atom with respect to the  remaining three. Especially the coordination of I1 resembles that of I in KIO_3_ where I is coordinated in a distorted octahedron (Lucas, 1984).

There are two strong hydrogen O—H···O bonds in the structure (Desiraju & Steiner, 1999) - see Tab. 1, Fig. 1. Moreover, there is also O-H···I interaction present in the structure (Tab. 1). Lepeshkov *et al.* (1977) report that dehydration takes place at 210°C, *i.e.* at quite a high temperature. Various sections from the title structure are depicted in Figs 1-5.

### Experimental

The title structure has been prepared by adding to 0.93 g of dissolved KIO_3_ in 20 ml of water of 0.4 g of KCl and 0.364 g of ZnCl_2_. The solution was heated up to 60 °C while adding water to 300 ml. A very fine precipitate has developed that did not dissolve completely. Fragile prism-like colourless crystals with length of several tenths of mm have developed in the course of three months. The crystals were twinned by a domain boundary perpendicular to the longer axis of the prism. The crystals that served for the measurement could be easily separated mechanically. However, these parts in some cases were not single-domained crystals. There seem to be other domain states as indicated measurement of several samples.

### Refinement

The water hydrogens have been detected in the difference electron density maps. It should be noted that they were observed with difficulties since close to O7 and O8 there have been other higher maxima situated precisely on the two-fold axis. The restraints were taken from the search in the Cambridge Structural Database (Allen, 2002). The search in the Database referred to the O—H distances and the angle H—O—H of the coordinated water molecules on Zn. The Database provided 1000 hits. The restrained values were: Zn—O—H = 125.50 (1)° and O—H = 0.845 (1) Å. The used constraints: U_iso_(H)=1.5U_eq_O. Moreover, because of the space group C2 the *y*-coordinate of I1 has been fixed.

### Crystal data

**Table:**

K_2_Zn(IO_3_)_4_·2H_2_O	*F*(000) = 792
*M**_r_* = 879.2	*D*_x_ = 4.112 (1) Mg m^−^^3^
Monoclinic, *C*2	Mo *K*α radiation, λ = 0.71069 Å
Hall symbol: C 2y	Cell parameters from 8075 reflections
*a* = 13.8044 (3) Å	θ = 2.9–27.5°
*b* = 7.7285 (2) Å	µ = 11.08 mm^−^^1^
*c* = 8.2860 (2) Å	*T* = 295 K
β = 126.5726 (13)°	Prism, colourless
*V* = 709.95 (3) Å^3^	0.17 × 0.12 × 0.05 mm
*Z* = 2	

### Data collection

**Table:**

Nonius KappaCCD area-detector diffractometer	1639 independent reflections
Radiation source: X-ray tube	1595 reflections with *I* > 3σ(*I*)
graphite	*R*_int_ = 0.045
Detector resolution: 9.091 pixels mm^-1^	θ_max_ = 27.5°, θ_min_ = 3.1°
φ and ω scans	*h* = −17→17
Absorption correction: gaussian (Coppens, 1970)	*k* = −10→10
*T*_min_ = 0.241, *T*_max_ = 0.581	*l* = −10→10
11909 measured reflections	

### Refinement

**Table:**

Refinement on *F*^2^	Hydrogen site location: difference Fourier map
*R*[*F*^2^ > 2σ(*F*^2^)] = 0.019	H atoms treated by a mixture of independent and constrained refinement
*wR*(*F*^2^) = 0.055	Weighting scheme based on measured s.u.'s *w* = 1/(σ^2^(*I*) + 0.0004*I*^2^)
*S* = 1.72	(Δ/σ)_max_ = 0.040
1639 reflections	Δρ_max_ = 0.60 e Å^−^^3^
105 parameters	Δρ_min_ = −0.61 e Å^−^^3^
4 restraints	Extinction correction: B-C type 1 Lorentzian isotropic (Becker & Coppens, 1974)
3 constraints	Extinction coefficient: 1040 (40)
Primary atom site location: structure-invariant direct methods	Absolute structure: Flack (1983), 761 Friedel pairs
Secondary atom site location: difference Fourier map	Flack parameter: −0.01 (3)

### Fractional atomic coordinates and isotropic or equivalent isotropic displacement parameters (Å^2^)

**Table:**

	*x*	*y*	*z*	*U*_iso_*/*U*_eq_	
K1	0.5	0.0000 (3)	0	0.0217 (9)	
K2	0.5	0.5290 (3)	0	0.0239 (10)	
I1	0.73832 (3)	0.270215	0.98624 (4)	0.01481 (16)	
I2	0.27889 (3)	0.28124 (7)	0.51786 (4)	0.01419 (16)	
Zn	0.5	0.45608 (17)	0.5	0.0173 (4)	
O1	0.8188 (3)	0.2594 (6)	0.8770 (5)	0.0199 (17)	
O2	0.3446 (3)	0.2678 (6)	0.7822 (5)	0.0230 (16)	
O3	0.6415 (4)	0.0814 (5)	0.8708 (5)	0.0220 (19)	
O4	0.1355 (4)	0.3798 (5)	0.4160 (6)	0.023 (2)	
O5	0.3581 (3)	0.4756 (5)	0.5308 (5)	0.0201 (18)	
O6	0.6246 (4)	0.4346 (5)	0.8313 (5)	0.0207 (18)	
O7	0.5	0.7158 (7)	0.5	0.039 (4)	
O8	0.5	0.1904 (7)	0.5	0.033 (3)	
H1O8	0.471 (6)	0.1269 (7)	0.3979 (19)	0.05*	
H1O7	0.478 (7)	0.7793 (7)	0.556 (10)	0.0591*	

### Atomic displacement parameters (Å^2^)

**Table:**

	*U*^11^	*U*^22^	*U*^33^	*U*^12^	*U*^13^	*U*^23^
K1	0.0231 (11)	0.0199 (9)	0.0240 (8)	0	0.0151 (8)	0
K2	0.0164 (10)	0.0199 (9)	0.0345 (10)	0	0.0148 (9)	0
I1	0.01408 (17)	0.0169 (2)	0.01324 (16)	0.0000 (2)	0.00804 (13)	0.00000 (15)
I2	0.01437 (18)	0.01408 (19)	0.01467 (16)	0.00005 (19)	0.00895 (13)	0.00057 (14)
Zn	0.0182 (5)	0.0151 (4)	0.0223 (4)	0	0.0142 (4)	0
O1	0.0197 (17)	0.0216 (19)	0.0229 (17)	0.0030 (18)	0.0152 (15)	0.0003 (16)
O2	0.0221 (18)	0.0275 (19)	0.0177 (16)	−0.005 (2)	0.0111 (14)	−0.0010 (18)
O3	0.022 (2)	0.0181 (18)	0.0195 (18)	0.0001 (17)	0.0090 (18)	0.0001 (15)
O4	0.017 (2)	0.024 (2)	0.031 (2)	0.0035 (15)	0.0160 (18)	0.0061 (15)
O5	0.0208 (19)	0.0138 (18)	0.0319 (19)	−0.0002 (15)	0.0191 (17)	−0.0002 (15)
O6	0.017 (2)	0.0214 (19)	0.0191 (17)	0.0056 (17)	0.0080 (16)	0.0004 (15)
O7	0.052 (4)	0.017 (3)	0.082 (5)	0	0.058 (4)	0
O8	0.041 (4)	0.016 (3)	0.024 (3)	0	0.009 (3)	0

### Geometric parameters (Å, °)

**Table:**

K1—O1^i^	2.778 (4)	I1—O6	1.823 (4)
K1—O1^ii^	2.778 (4)	I1—O6^xi^	3.031 (4)
K1—O2^iii^	2.744 (4)	I2—O1^iv^	2.701 (4)
K1—O2^iv^	2.744 (4)	I2—O2	1.807 (4)
K1—O3^iii^	2.802 (6)	I2—O4	1.795 (5)
K1—O3^iv^	2.802 (6)	I2—O4^xii^	3.248 (4)
K1—O4^v^	2.925 (4)	I2—O5	1.823 (4)
K1—O4^vi^	2.925 (4)	I2—O5^xii^	2.900 (4)
K2—O1^vii^	2.726 (4)	I2—O8	3.2202 (13)
K2—O1^viii^	2.726 (4)	Zn—O5	2.126 (6)
K2—O2^iii^	2.706 (4)	Zn—O5^iv^	2.126 (6)
K2—O2^iv^	2.706 (4)	Zn—O6	2.212 (3)
K2—O5^iii^	3.170 (4)	Zn—O6^iv^	2.212 (3)
K2—O5^iv^	3.170 (4)	Zn—O7	2.007 (6)
K2—O6^iii^	2.883 (6)	Zn—O8	2.053 (6)
K2—O6^iv^	2.883 (6)	O7—H1o7	0.85 (9)
I1—O1	1.805 (5)	O7—H1o7^iv^	0.85 (9)
I1—O2^ix^	2.758 (5)	O8—H1o8	0.845 (16)
I1—O3	1.818 (4)	O8—H1o8^iv^	0.845 (16)
I1—O3^x^	2.756 (4)		
			
H1o7—O7—H1o7^iv^	109 (6)	O5—Zn—O5^iv^	171.86 (15)
H1o8—O8—H1o8^iv^	109.0 (13)	O5—Zn—O6	86.97 (18)
O1^i^—K1—O1^ii^	95.96 (14)	O5—Zn—O6^iv^	93.64 (18)
O1^i^—K1—O2^iii^	94.78 (12)	O5—Zn—O7	85.93 (10)
O1^i^—K1—O2^iv^	157.90 (15)	O5—Zn—O8	94.07 (10)
O1^i^—K1—O3^iii^	133.68 (13)	O5^iv^—Zn—O6	93.64 (18)
O1^i^—K1—O3^iv^	67.05 (13)	O5^iv^—Zn—O6^iv^	86.97 (18)
O1^i^—K1—O4^v^	91.35 (12)	O5^iv^—Zn—O7	85.93 (10)
O1^i^—K1—O4^vi^	63.35 (14)	O5^iv^—Zn—O8	94.07 (10)
O1^ii^—K1—O2^iii^	157.90 (15)	O6—Zn—O6^iv^	171.39 (16)
O1^ii^—K1—O2^iv^	94.78 (12)	O6—Zn—O7	94.30 (11)
O1^ii^—K1—O3^iii^	67.05 (13)	O6—Zn—O8	85.70 (11)
O1^ii^—K1—O3^iv^	133.68 (13)	O6^iv^—Zn—O7	94.30 (11)
O1^ii^—K1—O4^v^	63.35 (14)	O6^iv^—Zn—O8	85.70 (11)
O1^ii^—K1—O4^vi^	91.35 (12)	O7—Zn—O8	180
O2^iii^—K1—O2^iv^	82.07 (13)	O1—I1—O2^ix^	169.37 (11)
O2^iii^—K1—O3^iii^	91.68 (14)	O1—I1—O3	100.2 (2)
O2^iii^—K1—O3^iv^	68.40 (13)	O1—I1—O3^x^	82.15 (18)
O2^iii^—K1—O4^v^	135.62 (14)	O1—I1—O6	102.0 (2)
O2^iii^—K1—O4^vi^	76.36 (11)	O1—I1—O6^xi^	79.79 (17)
O2^iv^—K1—O3^iii^	68.40 (13)	O1—I1—O8	81.25 (8)
O2^iv^—K1—O3^iv^	91.68 (14)	O2^ix^—I1—O3	83.2 (2)
O2^iv^—K1—O4^v^	76.36 (11)	O2^ix^—I1—O3^x^	95.72 (15)
O2^iv^—K1—O4^vi^	135.62 (14)	O2^ix^—I1—O6	87.4 (2)
O3^iii^—K1—O3^iv^	154.04 (14)	O2^ix^—I1—O6^xi^	92.39 (14)
O3^iii^—K1—O4^v^	114.81 (13)	O2^ix^—I1—O8	108.21 (6)
O3^iii^—K1—O4^vi^	73.91 (13)	O3—I1—O3^x^	172.60 (14)
O3^iv^—K1—O4^v^	73.91 (13)	O3—I1—O6	97.71 (16)
O3^iv^—K1—O4^vi^	114.81 (13)	O3—I1—O6^xi^	67.69 (14)
O4^v^—K1—O4^vi^	142.99 (13)	O3—I1—O8	49.04 (14)
O1^vii^—K2—O1^viii^	98.42 (15)	O3^x^—I1—O6	74.92 (14)
O1^vii^—K2—O2^iii^	92.96 (12)	O3^x^—I1—O6^xi^	119.70 (10)
O1^vii^—K2—O2^iv^	157.39 (15)	O3^x^—I1—O8	125.11 (11)
O1^vii^—K2—O5^iii^	82.40 (11)	O6—I1—O6^xi^	165.29 (13)
O1^vii^—K2—O5^iv^	107.60 (12)	O6—I1—O8	58.30 (14)
O1^vii^—K2—O6^iii^	131.60 (13)	O6^xi^—I1—O8	108.09 (11)
O1^vii^—K2—O6^iv^	70.54 (13)	O1^iv^—I2—O2	173.13 (18)
O1^viii^—K2—O2^iii^	157.39 (15)	O1^iv^—I2—O4	80.92 (18)
O1^viii^—K2—O2^iv^	92.96 (12)	O1^iv^—I2—O4^xii^	90.78 (12)
O1^viii^—K2—O5^iii^	107.60 (12)	O1^iv^—I2—O5	88.38 (16)
O1^viii^—K2—O5^iv^	82.40 (11)	O1^iv^—I2—O5^xii^	88.12 (13)
O1^viii^—K2—O6^iii^	70.54 (13)	O1^iv^—I2—O8	74.54 (9)
O1^viii^—K2—O6^iv^	131.60 (13)	O2—I2—O4	102.2 (2)
O2^iii^—K2—O2^iv^	83.50 (13)	O2—I2—O4^xii^	82.61 (17)
O2^iii^—K2—O5^iii^	54.54 (12)	O2—I2—O5	97.22 (19)
O2^iii^—K2—O5^iv^	112.69 (13)	O2—I2—O5^xii^	86.36 (17)
O2^iii^—K2—O6^iii^	87.30 (14)	O2—I2—O8	103.80 (15)
O2^iii^—K2—O6^iv^	70.70 (13)	O4—I2—O4^xii^	132.34 (15)
O2^iv^—K2—O5^iii^	112.69 (13)	O4—I2—O5	97.63 (19)
O2^iv^—K2—O5^iv^	54.54 (12)	O4—I2—O5^xii^	80.51 (16)
O2^iv^—K2—O6^iii^	70.70 (13)	O4—I2—O8	151.77 (18)
O2^iv^—K2—O6^iv^	87.30 (14)	O4^xii^—I2—O5	129.17 (17)
O5^iii^—K2—O5^iv^	165.03 (13)	O4^xii^—I2—O5^xii^	52.21 (12)
O5^iii^—K2—O6^iii^	58.89 (11)	O4^xii^—I2—O8	62.61 (14)
O5^iii^—K2—O6^iv^	116.79 (11)	O5—I2—O5^xii^	176.26 (13)
O5^iv^—K2—O6^iii^	116.79 (11)	O5—I2—O8	68.31 (19)
O5^iv^—K2—O6^iv^	58.89 (11)	O5^xii^—I2—O8	111.94 (14)
O6^iii^—K2—O6^iv^	150.67 (15)		
			
?—?—?—?	?		

Symmetry codes: (i) *x*−1/2, *y*−1/2, *z*−1; (ii) −*x*+3/2, *y*−1/2, −*z*+1; (iii) *x*, *y*, *z*−1; (iv) −*x*+1, *y*, −*z*+1; (v) *x*+1/2, *y*−1/2, *z*; (vi) −*x*+1/2, *y*−1/2, −*z*; (vii) *x*−1/2, *y*+1/2, *z*−1; (viii) −*x*+3/2, *y*+1/2, −*z*+1; (ix) −*x*+1, *y*, −*z*+2; (x) −*x*+3/2, *y*+1/2, −*z*+2; (xi) −*x*+3/2, *y*−1/2, −*z*+2; (xii) −*x*+1/2, *y*−1/2, −*z*+1.

### Hydrogen-bond geometry (Å, °)

**Table:**

*D*—H···*A*	*D*—H	H···*A*	*D*···*A*	*D*—H···*A*
O7—H1O7···O4^xiii^	0.85 (9)	1.88 (10)	2.672 (6)	155 (5)
O7—H1O7^iv^···O4^xiv^	0.85 (9)	1.88 (10)	2.672 (6)	155 (5)
O8—H1O8···O3^iv^	0.845 (16)	1.837 (16)	2.610 (4)	151 (4)
O8—H1O8···I1^iv^	0.845 (16)	2.96 (3)	3.4119 (10)	116 (3)

Symmetry codes: (xiii) −*x*+1/2, *y*+1/2, −*z*+1; (iv) −*x*+1, *y*, −*z*+1; (xiv) *x*+1/2, *y*+1/2, *z*.
